# A differential impact of action–effect temporal contiguity on different measures of response inhibition in the Go\No-Go and Stop-signal paradigms

**DOI:** 10.1007/s00426-024-01931-2

**Published:** 2024-03-07

**Authors:** Noam Karsh, Eden Soker-Mijalevich, Omer Horovitz

**Affiliations:** 1 Department of Psychology, Tel-Hai Academic College, Upper Galilee, Qiryat Shemona, Israel; 2 Special Population Advance Research and Clinical Center (SPARC), University of Haifa, Haifa, Israel

## Abstract

Response inhibition refers to suppressing a prepotent motor response and is often studied and discussed as an act of cognitive control. Much less attention was given to the potential contribution of motor control processes to response inhibition. Accumulated empirical findings show that a perceptual effect temporally contiguous with a response improves motor control performance. In the current study, we followed this work by manipulating action–effect temporal contiguity to enhance motor performance and investigated its impact on response selection and inhibition. In two experiments, we integrated a Go/No-Go (GNGT; Experiment 1) and a Stop-signal (SST; Experiment 2) task with the Effect–Motivation task, previously used to capture the facilitating impact of action–effect temporal contiguity on response times (RTs). Replicating previous findings, RTs were shorter following temporally contiguous compared to Lagged action–effect in Go trials in both the GNGT (Experiment 1) and SST (Experiment 2). Notably, an Immediate action–effect improved response inhibition in the GNGT (Experiment 1) but did not modulate Stop-signal reaction time (SSRT) in the SST (Experiment 2). Unexpectedly, the error rate on Go trials was higher in the Immediate effect condition in Experiment 2. We interpret the findings to suggest that an action’s (Immediate) perceptual effect may promote response inhibition performance by enhancing selective association between the Go stimuli and the Go response and not by improving cognitive control ability. The findings also imply that an Immediate action–effect may hamper action control (e.g., by increasing general readiness to respond), at least when action control does not benefit from automatic stimulus–response association.

**Supplementary Information:**

The online version contains supplementary material available at 10.1007/s00426-024-01931-2.

## Introduction

Response inhibition is a fundamental ability to withhold a prepotent response or stop an ongoing response (Barkley, 1999). Response inhibition is crucial for regulating behavior according to social norms and goal-directed behavior and preventing adverse outcomes from executing impulsive actions (Bari & Robbins, 2013). Deficits in response inhibition ability were observed in psychopathological and neurological disorders, such as schizophrenia (Enticott et al., 2008), attention-deficit/hyperactivity disorder (ADHD, e.g., Wodka et al., 2007), compulsive disorders (Penadés et al., 2007), and autism (Geurts et al., 2014).

Response inhibition is commonly described as an act of cognitive control or executive function that enables individuals to adapt rapidly to environmental changes (Barkley, 1997; Schachar et al., 2000). Previous research on response inhibition has primarily focused on developing cognitive abilities, such as the association between response inhibition, working memory, and attention (for review, see Chambers et al., 2009).

Comparatively less research focused on the potential contribution of motor control to response inhibition (for a relevant review, see Mostofsky & Simmonds, 2008). Refining motor processes and successful response inhibition can be attributed to motor experience and expertise (Mann et al., 2007; Molina et al., 2019; Zhang & Rowe, 2014). For instance, Simpson et al. (2019) found that fine motor control was associated with inhibition, statistically explaining drawing skills. Similarly, developmental research has shown that response execution and inhibition improve throughout childhood (Williams et al., 1999).

The link between motor control performance and response inhibition is also observed in neurodevelopmental disorders (Diamond, 2000). Individuals diagnosed with attention deficit\hyperactivity disorder (ADHD) often exhibit poor motor coordination and are prone to act impulsively (Wodka et al., 2007). Similarly, individuals diagnosed with poor motor control, such as developmental coordination disorder (DCD), tend to perform poorly on tasks that require response inhibition (Leonard et al., 2015). Although the link between motor performance and response inhibition is commonly observed, it is still poorly understood.

As elaborated next, previous work investigating the contribution of an own-action’s sensory effect to sensorimotor processes and motor control demonstrated its enhancing impact on motor performance (Eitam et al., 2013; Hemed et al., 2020; Karsh & Eitam, 2015a, 2015b; Karsh et al., 2016, 2020, 2021, 2023; Tanaka et al., 2021). We built on this work to investigate the link between motor enhancement by action–effect and response inhibition.

### The impact of action’s perceptual effect on motor performance

An own-action–effect plays a vital role in motor control and sensorimotor processes (Elsner & Hommel, 2004; Miall & Wolpert, 1996; Wolpert et al., 1995, 2011). Notably, a perceptual effect must first be registered as an own-action–effect to impact motor processes. A motor-based computational process, known as the comparator model, was suggested to be responsible for evaluating the causal relationship between a motor action and a perceptual effect (Frith et al., 2000; Synofzik et al., 2008). According to this model, motor-based sensory predictions are compared with the representations of the actual sensory feedback. A discrepancy between the predicted and the actual sensory feedback is computed and evaluated at a pre-conceptual sensorimotor level, and it is susceptible to physical properties of the perceptual effect, such as its temporal contiguity with the response (Blakemore et al., 1999, 2002; Frith et al., 2000; Wen, 2019; Wen & Imamizu, 2022). According to this model, a perceptual effect must be temporally contiguous with the response to be registered by the motor system as an own-action–effect.

Focusing on the motor outcomes of this process, previous work documented the promoting impact of an Immediate action–effect on the speed and precision of motor behavior (e.g., Eitam et al., 2013; Karsh et al., 2016, 2023). For instance, in a speeded reaction time task, a perceptual effect (a brief white flash) that follows immediately after the response (compared to a delayed action–effect, spatially unpredicted and No-effect) was repeatedly demonstrated to facilitate response times (Eitam et al., 2013; Karsh & Eitam, 2015a, 2015b; Karsh et al., 2016; Penton et al., 2018; Hemed et al., 2020; Karsh et al., 2020; Tanaka et al., 2021; Hemed et al., 2022). Notably, the facilitating impact of an Immediate (compared to Lagged) action–effect on response times was independent of participants’ attentional engagement in the task (Karsh et al., 2016) and the probability of the effect when the motor system was not involved (Hemed et al., 2022). Accordingly, response facilitation in these tasks was suggested to result from the reinforcing impact of an Immediate action–effect on motor response selection processes (Hemed et al., 2022; Karsh & Eitam, 2015b). Such facilitation effect was recently demonstrated to depend on a specific combination between the stimulus and the response (Tanaka et al., 2021), indicating a promotive impact of an Immediate action–effect on the development of stimulus–response associations (e.g., Paulus et al., 2012; Tanaka et al., 2021).

The above studies indicate that an Immediate perceptual effect that follows a response (compared to a subtly Lagged action–effect) facilitates the selection of the relevant responses, possibly by enhancing the development of the stimulus–response association. However, whether and how it affects response inhibition is unclear. Investigating this question will advance our understanding of the link between motor control performance and response inhibition, which may provide important implications for understanding action control challenges.

### How temporally contiguous action–effect may impact response inhibition

The Go/No-Go (GNGT) and the Stop-signal tasks (SST) are commonly used for measuring response inhibition, yet growing evidence suggests that they recruit different mechanisms (Dambacher et al., 2014; Eagle et al., 2008; Raud et al., 2020; Schachar et al., 2007; Sebastian et al., 2013; Swick et al., 2011; Verbruggen & Logan, 2008, 2009). In typical GNGT, participants are instructed to respond in trials where a frequent Go stimulus is presented and occasionally withhold their response in trials where a No-Go stimulus appears instead of the Go stimulus. Response inhibition performance in the GNGT can be calculated by the proportion of Go responses in No-Go trials. Unlike the GNGT, in the SST, the Go stimulus is always presented at the beginning of the trial, and the Stop signal occasionally follows the Go signal after a variable delay. Response inhibition in the SST can be measured by the Stop-signal response time (SSRT), which reflects the latency of the covert Stop process. SSRT can be calculated by subtracting the averaged Stop-signal delay (SSD), which indicates the time delay between the Go signal and the Stop signal at which the participant has a 50% chance of stopping their response from the averaged Go-RT.

Such methodological differences recruit different processes. In the GNGT, an automatic association can be developed between the Go stimulus and the Go response and between the No-Go stimuli and the No-Go response and further contribute to response inhibition without additional cognitive control resources (Schachar et al., 2007; Schneider & Shiffrin, 1977; Verbruggen & Logan, 2008). In contrast, such an automatic association is less likely to develop in the SST because the Go stimulus is non-selectively presented in both the Go and Stop trials.

For example, investigating the contribution of automatic stimulus–response (S–R) association to response inhibition in the GNGT, Verbruggen and Logan (2008) asked participants to perform a semantic judgment (e.g., living or non-living). The requirement for a response was determined by the stimulus category (e.g., living as go and non-living as no-go). After a training phase where the S–R association was developed, participants performed a test phase where the Go and No-Go categories were consistent or inconsistent (e.g., non-living as go and living as no-go) with the training phase. Supporting the contribution of automatic S–R association to performance, in the inconsistent condition, RT was slower for stimuli associated with No-Go in the training phase (compared to new stimuli). In addition, in the consistent condition, success in no-go performance was higher for stimuli also appearing in the training phase (compared to new stimuli). These findings demonstrated the contribution of S–R association to automatic inhibition in the GNGT.

As indicated above, the S–R association is less likely to contribute to automatic inhibition in the SST where the Go stimulus appears in both the Go and Stop trials. Response inhibition in the SST is commonly attributed to cognitive control and explained by the influential horse-race model for response inhibition (Logan & Cowan, 1984; Verbruggen & Logan, 2008, 2009; Bissett et al., 2009; Littman & Takacs, 2017). The model proposes that response inhibition is determined by the outcome of a competition between two independent processes: a motor response activation and a cognitive response inhibition. In this model, faster responses are more likely to “win” the competition and escape inhibition.

In the context of the current study, a previous work by Tanaka et al. (2021) suggests that an Immediate action–effect enhances stimulus–response association. Specifically, the authors developed an adapted version of the ‘Effect-Motivation’ task (Eitam et al., 2013) to investigate whether an Immediate (compared to 600 ms Lagged) action–effect reinforces the specific response, the stimulus, or stimulus–response association. Their findings demonstrated that the facilitating impact of an Immediate compared to Lagged action–effect on the speed of response selection depends on a particular combination between the stimulus and the response, potentially reinforcing the stimulus–response association and contributing to the refinement of sensorimotor representations.

Important for the current study, such enhanced stimulus–response association may also contribute to automatic inhibition in the Immediate (compared to Lagged) effect conditions. For instance, an improved selective association between the Go stimulus and the Go response may facilitate Go responses following the Go stimulus and reduce the probability of Go responses to other stimuli (e.g., the No-Go stimulus), contributing to a higher rate of successful inhibitions in the GNGT.

Preliminary evidence that an Immediate action–effect contributes to No-Go performance comes from our unpublished study using a free-choice paradigm where participants performed a modified version of a speeded reaction time task (Eitam et al., 2013). Participants were required to respond as fast as possible to descending circles on their computer monitor. In some trials, a rectangle occasionally replaced the circle Go stimuli, indicating that participants should decide ‘freely' whether to respond in that trial. Consistent with previous studies (e.g., Eitam et al., 2013; Karsh et al., 2016), their RTs on Go trials were shorter when an Immediate (compared to 450ms delayed) effect followed their responses. Intriguingly, the proportion of their No-Go behavioral decisions was nominally higher in the Immediate compared to the Lag effect condition. Although this study was not designed to measure response inhibition directly, it empirically motivated the present study’s predictions that temporally contiguous action–effect will facilitate the speed of responses and increase the rate of successful No-Go trials.

Notably, previous theoretical and empirical accounts suggest minimal involvement of non-motor and controlled processes (e.g., monetary incentives, general motivation to succeed in the task, and conscious considerations; Eitam et al., 2013; Karsh & Eitam., 2015a, b; Karsh et al., 2016; Tanaka et al., 2021; Karsh et al., 2020; Hemed et al., 2022) in the facilitating impact of an action’s immediate effect on RT. Therefore, we expected action–effect temporal contiguity manipulation in the current study will tap into sensory and motor processes with a minimal impact on controlled processes (e.g., Hemed et al., 2022; Karsh et al., 2020). Accordingly, we hypothesized an Immediate action–effect would facilitate Go response speed and improve automatic inhibition performance in the GNGT but not inhibitory control performance (SSRT) in the SST. We integrated the GNGT (Experiment 1) and SST (Experiment 2) with the Effect–Motivation task (Eitam et al., 2013), previously established to measure the impact of action–effect temporal contiguity on RTs. In both experiments, action–effect temporal contiguity (Immediate vs. 450 ms Lagged action–effect) was manipulated in different blocks in a within-subject design. Consistent with previous studies (Eitam et al., 2013; Karsh et al., 2016; Tanaka et al., 2021), we expected RTs in Go trials would be shorter in the Immediate compared to the Lag effect condition in both tasks. Importantly, we also expected a better No-Go performance in No-Go trials in the Immediate compared to Lagged effect conditions (Experiment 1) and that SSRT would not be affected by action–effect temporal contiguity in the SST (Experiment 2). However, we expected the average SSD to be smaller in the Immediate than in the Lag effect condition, reflecting the race between the Go and Stop processes (see pre-registration: https://osf.io/dc93t/?view_only=14f9f30baeb1462b927121e155222186).

## Experiment 1: The impact of action–effect temporal contiguity on performance in a Go\No-Go task

### Method

#### Participants

Eighty-three students from the Psychology Department at Tel-Hai Academic College participated in the study in exchange for course credit. We continued to collect data until we reached 71 valid participants. The required sample size was calculated using GPOWER software (Erdfelder et al., 1996) to achieve an ~ 80% chance of detecting a conservative effect size (dz = 0.3) in a one-tailed comparison between two paired means (see Data analysis section for more details). The expected effect size was determined based on our previous unpublished study (https://osf.io/w375z/?view_only=e30192d667264a9ba865739973bcb81c) for the difference in the proportion of No-Go responses between conditions. The final sample comprised 71 participants. Among them, 43 were identified as females, and 28 were identified as males (Age: *M* = 25.07, SD = 2.94). The Institutional Review Board Committee of Tel-Hai Academic College approved the experiments (ref. 05\2018–4).

#### Stimuli and procedure

**Fig. 1:**
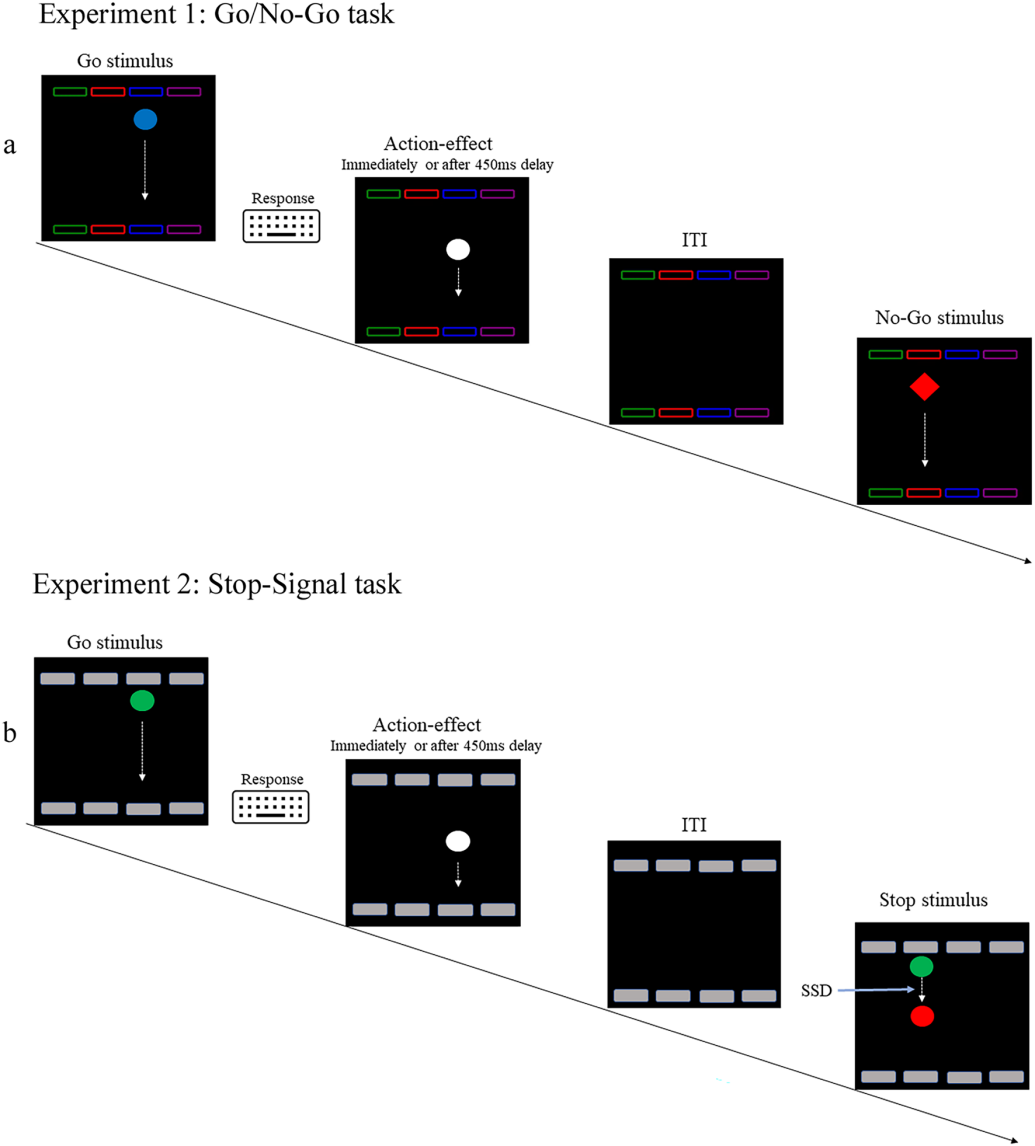
A simulation of the **a** Go\No-Go (Experiment 1) and the **b** Stop-signal tasks (Experiment 2) in the current study

Participants were invited to the lab and completed an informed consent form. Participants were then sat in cubicles with dim lighting before a computer monitor (60 Hz, AOC monitor, 53cm wide) and introduced to a modified version of the Effect–Motivation task (Eitam et al., 2013). In each trial, a colored circle (green, red, blue, or purple; 53 pixels in diameter, see Fig. 1a) appeared randomly in one of four possible horizontal locations at the top of the game window (9.5 cm horizontal X 10 cm vertical). The colored circle rapidly descends vertically to the bottom of the game window (~ 57 cm from the participant) for 1100 ms (if no response was registered). Importantly, ISI was 1300 ms, regardless of participants’ RT.

Participants were to place both the middle and index fingers of both hands on four response keys (‘S,’ ‘D,’ ‘H,’ and ‘J’) on a standard PC keyboard. They were instructed to ‘stamp’ the circles (the Go stimuli) as they appeared on the screen as fast as possible by pressing the relevant (spatially and color-coded) key. A perceptual effect (a brief flash) appeared after participants’ responses (the circle changes to white for 100 ms and disappears). Notably, the perceptual effect immediately followed the participant’s response or lagged by 450 ms.

Participants performed both temporal contiguity (Immediate and 450 ms delay) conditions sequentially in separate blocks (180 trials for each condition) in a counterbalanced order. Notably, we included No-Go trials in which a square replaced the circle in 20% of the trials (36 trials for each temporary contiguity condition block, 72 No-go trials overall). Participants were instructed to avoid responding on No-Go trials.

At the end of the task, we asked the participants to report their subjective experience in the task, including their invested effort, mood, the importance of succeeding in the task, the importance of being accurate, the importance of responding fast, their felt urge to generate the effect, and their enjoinment level on a scale ranging from 0 (not at all) to 100 (very much).

#### Data analysis

We applied a filter similar to our previous study (Karsh et al., 2020) and followed our unpublished study report. First, we removed participants with less than two standard deviations in their proportion of inhibitions in at least one of the experimental conditions (11 participants) and participants with less than 80% correct responses in the Go trials (one participant). Next, we calculated the participants’ proportion of correct responses in Go trials in each condition. Finally, trials with incorrect responses or misses in the Go trials (~ 4% of the remaining trials) were removed before generating the Go-RT variable. Consistent with our estimated power calculation, we report one-tailed paired-sample *t* tests for the differences in Go-RT and the proportion of inhibitions in the Immediate compared to the Lag effect condition. For all other tests, we report the two-tailed *p* value. In addition to null hypothesis testing, to evaluate the sensitivity of the data in supporting the alternative or the null hypothesis, we also computed Bayesian paired-samples *t* test using Cauchy prior; width = 0.707 (JASP Team, 2023) and reported the Bayes Factor (BF).

### Results and discussion

**Fig. 2:**
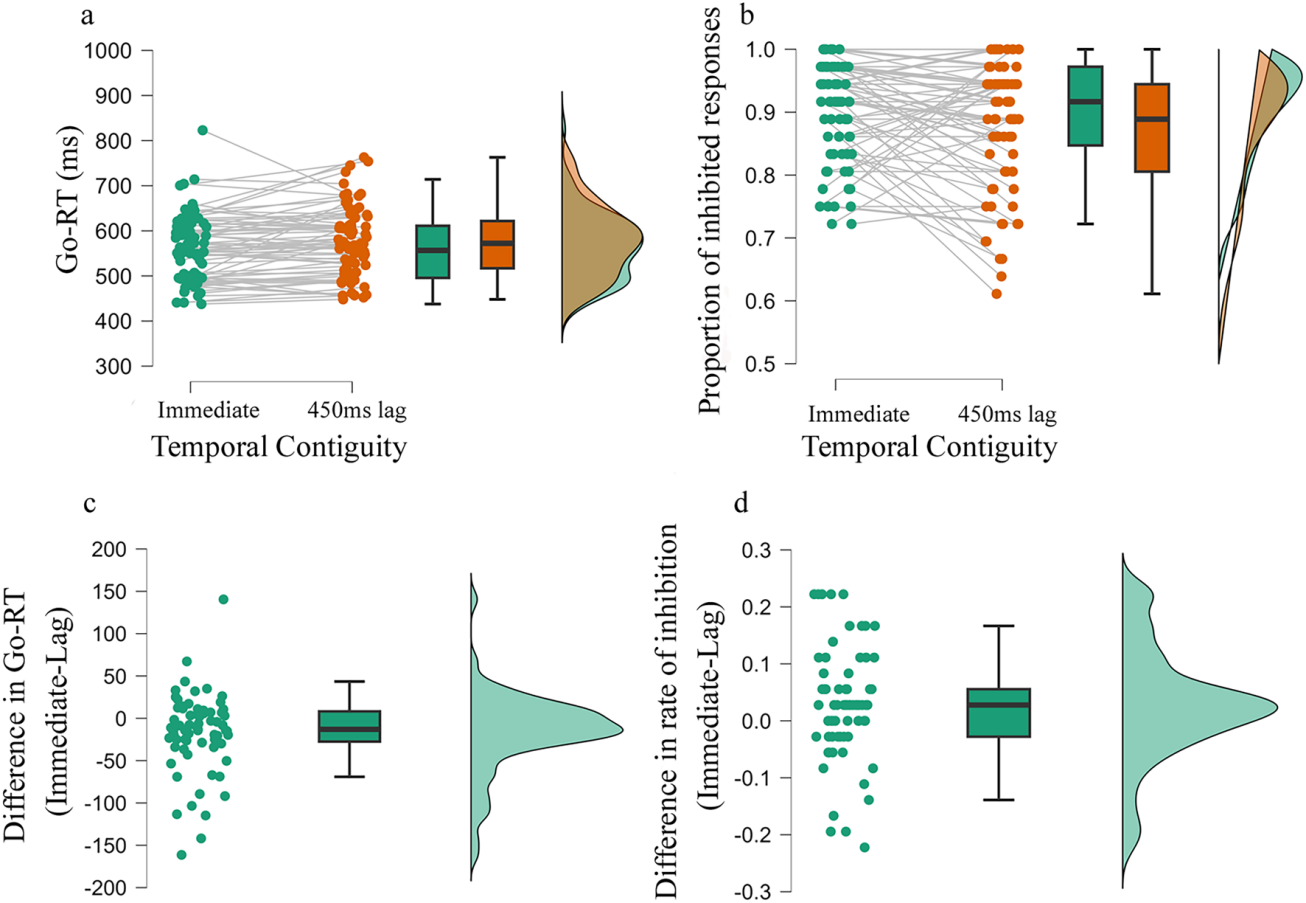
The impact of action–effect temporal contiguity on performance in Experiment 1. The figures present a raincloud plot including individual data points, a box plot, and a one-sided violin plot of **a** response time in Go trials, **b** proportion of successful No-Go trials, **c** response time difference between conditions in Go trials, and **d** difference in the proportion of inhibited responses between conditions

Replicating previous studies (Eitam et al., 2013; Karsh & Eitam, 2015a, 2015b; Karsh et al., 2016; Hemed et al., 2020; Karsh et al., 2020; Tanaka et al., 2021), Go-RT was shorter in the Immediate (*M* = 562, SD = 74) compared to the Lag (*M* = 578, SD = 77) effect condition [*t*_70_ = 2.98, *p* = 0.002, CI_95_ (5, 27), *dz* = 0.35, BF_10_ = 14.8; Fig. 2a and 2b]. In addition, the proportion of correct responses in Go trials was only nominally higher [*t*_70_ = 1.9, *p* = 0.06, CI_95_ ( – 0.0, 0.01), *dz* = 0.22, BF_10_ = 1.3] in the Immediate (*M* = 0.94, SD = 0.03) compared to the Lag (*M* = 0.93, SD = 0.04) effect condition. No significant correlation was observed between the rate of correct Go trials and Go-RT in the Immediate (*r* = 0.02, *p* = 0.81, BF_10_ = 0.15) and the Lag (*r* = -0.14, *p* = 0.21, BF_10_ = 0.31) conditions.

Critically, the rate of successful No-Go trials was higher in the Immediate (*M* = 0.9, SD = 0.07) compared to the Lag (*M* = 0.87, SD = 0.1) effect condition [*t*_70_ = 2.38, *p* = 0.009, CI_95_ (0.004, 0.05), *dz* = 0.28, BF_10_ = 3.63; Fig. 2c and 2d] suggesting that temporally contiguous action–effect contributed to response inhibition without the cost of slowing Go responses.

Regardless of such between-conditions differences, Go-RT was positively correlated with the rate of No-Go responses in both the Immediate (*r* = 0.31, *p* = 007; BF_10_ = 5.2) and the Lag (*r* = 0.3, *p* = 01; BF_10_ = 3.5) conditions. Such correlation reflects a cognitive response strategy to trade speed in Go trials for accuracy in No-Go trials. Critically, the difference between action–effect temporal contiguity conditions emerged above and beyond this tradeoff.

Finally, exploring our self-report indicators for general motivational engagement\resource allocation to the task, the proportion of inhibited responses was negatively correlated with participants’ subjective importance to respond fast in the Immediate [*r* =  – 0.26, *p* = 0.02, BF_10_ = 1.67] but not in the Lag (*r* =  – 0.19, *p* = 0.1, BF_10_ = 0.5) effect condition. However, these correlations were not statistically different (*z* =  – 0.56, *p* = 0.28), possibly reflecting an explicit knowledge of participants’ response strategy. No other self-report measures significantly correlated with the proportion of inhibited responses (see Table S1 for a full correlational matrix).

Experiment 1 confirmed our predictions by providing substantial evidence that the temporally contiguous action–effect improves motor performance, as indicated by shorter Go-RT and a higher rate of successful No-Go trials. Such enhanced performance cannot be explained by general readiness to respond or modulation of response threshold following Immediate action–effect.

Following the hypothesis that the contribution of temporally contiguous action–effect operates at the sensorimotor (e.g., enhancing selective association between the Go stimulus and the Go response; e.g., Tanaka et al., 2021) rather than the cognitive control level, in Experiment 2, we directly investigated the impact of action–effect temporal contiguity on cognitive control performance.

Specifically, in Experiment 2, we developed a novel task, integrating the SST with the Effect–Motivation task (Eitam et al., 2013), and examined the impact of action–effect temporal contiguity on Go-RT and the Stop-signal RT (SSRT). Because the Go stimulus appears on both Go and Stop trials in the SST, automatic processes have a limited effect on response inhibition (Verbruggen & Logan, 2008). Thus, cognitive control ability plays a prominent role in modulating SSRT. According to the above, we expected the temporally contiguous action–effect to facilitate Go-RTs in the frequent Go trials without modulating Stop-signal reaction times (SSRT).

## Experiment 2: the impact of action–effect temporal contiguity on performance in a Stop-signal task

### Method

#### Participants

Thirty-three students from Tel-Hai Academic College were recruited to participate in the study. We aimed to collect data from 32 participants, which is expected to yield a 90% chance of detecting a significant difference in a two-tailed paired-sample *t* test with an estimated dz = 0.6 effect size. Previous studies showed that the effect size corresponding to the facilitating impact of temporally contiguous action–effect ranges from dz =  ~ 0.35 (in Experiment 1) to ~ 0.96 (Karsh et al., 2016). However, the effect size for the difference in the proportion of inhibited responses in the Go\No-Go task in Experiment 1 was small. Thus, because of this and the fact that we expect no difference in SSRT between conditions, our stopping rule was determined based on a conclusive Bayes Factor supporting the alternative (BF_10_ > 3) or the null (BF_10_ < 0.3) hypothesis (Dienes, 2014, 2016; Schönbrodt et al., 2017) for all three predictions. Bayesian *t* test was calculated in JASP (JASP Team, 2023) software using the default Cauchy prior; width = 0.707. After data from one participant were removed from the analysis (see the Data pre-processing section for more information), the final sample comprised 32 participants. Among them, 23 were identified as females, and 9 were identified as males (Age: *M* = 25.9, SD = 1.69).

#### Stimuli and procedure

Participants were invited to the lab, sat in individual cubicles with dim lighting before a computer monitor, and were introduced to the task (Fig. 1b). In this task, a green circle (Go signal; 2 cm in diameter) appears in one of four possible horizontal locations at the top of the game window (14cm X 11.5 cm). The circle rapidly descended vertically (for 1300 ms or until response) to the bottom of the game window (located about 57 cm from the participant, ITI = 0.2). Participants placed both hands’ middle and index fingers on four designated response keys (‘S,’ ‘D,’ ‘H,’ and ‘J’) on a standard PC keyboard. Participants were instructed to ‘stamp’ the green circles as they appeared on the screen as fast as they could by pressing the relevant (spatially coded) key. A perceptual effect followed participants’ responses (the circle changes to white for 100 ms and disappears, experienced as a brief flash). Notably, in a counterbalanced order, action–effect temporal contiguity was manipulated in separate blocks for each participant. Specifically, the perceptual effect could appear immediately or 450 ms after the response (ISI = 1500 ms, regardless of participants' RT). In 25% of the trials (60 trials for each temporal contiguity condition), the green Go signal changed its color to a red (Stop signal) after an adjusted delay (Stop-signal delay; SSD). SSD was adjusted after Stop trials using the tracking procedure. Baseline SSD was set to 300 ms at the beginning of each condition and increased after successful or decreased after unsuccessful stopping in steps of 50 ms. This procedure was expected to yield about 50% inhibition rate. SSRT was calculated by the mean method, subtracting mean SSD from mean RT in the Go trials.

After a short practice session, participants performed 240 trials of each condition block, with a few minutes break between blocks. Go and No-Go trials were randomly selected (without replacement) from a pre-specified list by the computer. At the end of the task, we probed participants’ invested effort, enjoyment, the importance of succeeding in the task, and their felt urge to generate the effect on a scale ranging from 1 (not at all) to 7 (very much).

#### Data analysis

The following filter was applied according to the pre-registration (https://osf.io/dc93t/?view_only=14f9f30baeb1462b927121e155222186). Participants with less than 80% correct responses (one participant) were removed before generating the proportion of correct responses on the Go trials variable. Next, incorrect responses on Go trials (~ 5%) and responses on the Go trials (~ 4% of the remaining trials) that are too slow (RT > 700 ms) or too fast (RT < 200 ms) were removed from the analyses before generating the remaining dependent variables.

### Results and discussion

First, reflecting the nature of the task, participants were able to stop their prepotent responses in about 50% of the trials (Immediate: min = 0.45, max = 0.56; Lag: min = 0.46, max = 0.51) and there was no significant difference in the proportion of stopping between the Immediate (*M* = 0.48, SD = 0.02) and the Lag (.*M* = 0.49, SD = 0.01) effect condition [*t*_31_ = 0.94, *p* = 0.17, CI_95_ ( – 0.004, 0.01), *dz* = 0.16, BF_10_ = 0.28].

Surprisingly, the proportion of correct responses on Go trials was significantly lower in the Immediate (*M* = 0.92, SD = 0.05) compared to the Lag (*M* = 0.94, SD = 0.03) effect condition [*t*_31_ = 2.32, *p* = 0.026, CI_95_ (0.001, 0.02), *dz* = 0.41, BF_10_ = 1.93]. Notably, the proportion of correct Go trials did not significantly correlate with SSRT in the Immediate (*r* =  – 0.15, *p* = 0.4, BF_10_ = 0.3) and the Lag (*r* =  – 0.3, *p* = 0.08, BF_10_ = 0.8) effect conditions.

**Fig. 3:**
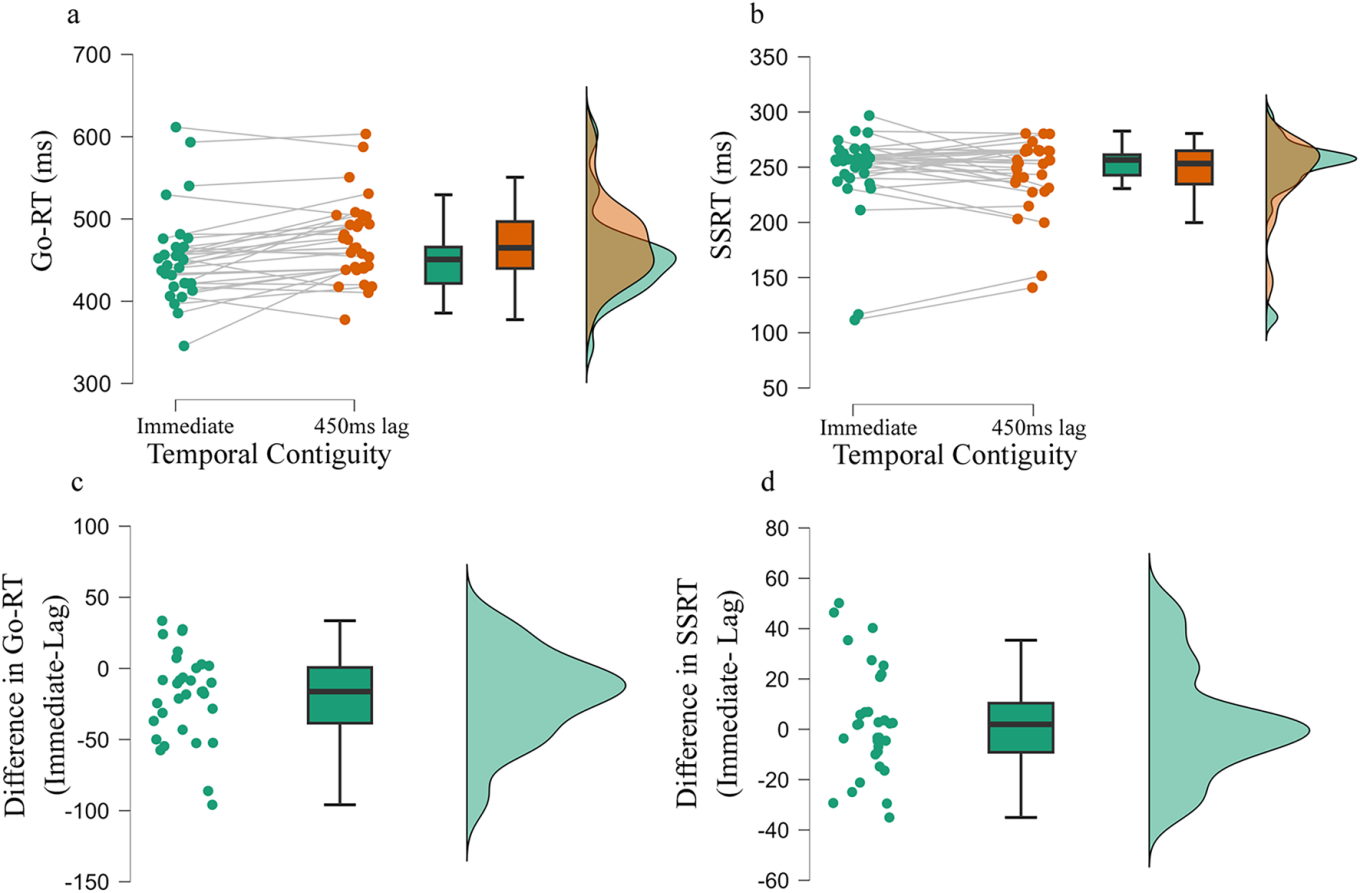
The impact of action–effect temporal contiguity on performance in Experiment 2. The figures present a raincloud plot including individual data points, a box plot, and a one-sided violin plot of **a** response time in Go trials, **b** Stop-signal reaction time (SSRT), **c** response time difference between conditions in Go trials, and **d** difference in SSRT between conditions

Replicating previous studies (e.g., Eitam et al., 2013; Karsh et al., 2016; Tanaka et al., 2021), Go-RT was shorter in the Immediate (*M* = 453, SD = 54) compared to the Lag (*M* = 472, SD = 49) effect condition [*t*_31_ = 3.52, *p* < 0.001, CI_95_ (8, 30), *dz* = 0.62, BF_10_ = 24.95; Fig. 3a and 3b]. To further explore whether differences in the proportion of correct responses can explain the impact of action–effect delay on Go-RT, we regressed both action–effect temporal contiguity and the proportion of correct responses on Go-RT, clustered by participants (*R*^2^ = 0.11, *p* < 0.001). Both temporal contiguity (Coef. = 0.03, SE = 0.01, *p* = 0.02) and the proportion of correct responses on Go trials (Coef. = 337, SE = 139, *p* = 0.02) were statistically significant, indicating a distinct contribution of action–effect temporal contiguity and proportion of correct responses in predicting Go-RT.

Consistent with our predictions, SSD was smaller (*M* = 206, SD = 84) in the Immediate compared to the Lag (*M* = 228, SD = 73) effect condition [*t*_31_ = 3.15, *p* = 0.001, CI_95_ (7, 36), *dz* = 0.55, BF_10_ = 10.63], reflecting the competition between the Go and Stop processes. Notably, the facilitating impact of the temporally contiguous action–effect on the speed of responses was not accompanied by a significant change in SSRT, which did not statistically differ between the Immediate (*M* = 246, *SD* = 38) and the Lag (*M* = 243, SD = 32) effect condition [*t*_31_ = 0.7, *p* = 0.24, CI_95_ ( – 10, 5), *dz* = 0.12, BF_10_ = 0.23]. No significant correlations were observed between the behavioral and the self-report measures (see Fig. S2 for a full correlational matrix).

### General discussion

Response inhibition involves controlling motor impulses or suppressing prepotent responses, and thus plays a vital role in goal-directed behavior. Extensive work on response inhibition ability focused on inhibitory control and executive functions (Baddeley, 1998; Chambers et al., 2009; Ridderinkhof et al., 2004), while the potential contribution of motor control to response inhibition was relatively unexplored.

In the current study, we followed theoretical and empirical progress in understanding the promoting impact of a temporally contiguous perceptual effect on the speed and precision of motor performance (e.g., Eitam et al., 2013; Karsh et al., 2016, 2023) to directly investigate its potential contribution to response inhibition. For this purpose, we developed a modified version of GNGT and SST and manipulated the temporal contiguity between a Go response and its perceptual consequence (action–effect).

Consistent with our predictions and replicating previous work (Eitam et al., 2013; Karsh et al., 2016), temporally contiguous (compared to 450 ms Lagged) action–effect facilitated the speed of Go responses in both the GNGT (Experiment 1) and the SST (Experiment 2). Critically consistent with our predictions, Experiment 1 revealed that an enhanced No-Go performance in the GNGT accompanied response facilitation in Go trials. This pattern reflects a superior performance above and beyond the typical cognitive strategy to trade speed for accuracy. Thus, a simple mechanism in which temporally contiguous action–effect increases general readiness to respond or modulation of response threshold cannot explain the difference in response inhibition between conditions in the GNGT.

Our self-report indicators for general motivation or resource allocation to the task were not significantly associated with the proportion of No-Go responses. If anything, the more participants tried to be faster, the lower the success in No-Go trials. It should be noted that the explicit reports were measured once at the end of the experimental task, and thus may not be sufficiently sensitive to capture potential differences in explicit reports between conditions.

Finally, in Experiment 2, we measured the Stop-process latency (SSRT), which is highly affected by executive or cognitive control resources. The findings confirmed that the temporally contiguous action–effect substantially facilitated the speed of motor responses on Go trials critically without affecting SSRT. Surprisingly, the findings of Experiment 2 also revealed a higher error rate in Go trials and a lower rate of successful stopping (after excluding two outliers; see Supplemental Materials) in the Immediate (compared to the Lag) effect condition in the SST.

#### How does the temporally contiguous action–effect influence response inhibition?

Action–effect was suggested to have a rewarding impact that further reinforces response selection and enhances stimulus–response association when it is temporally contiguous with a response (Hemed et al., 2022; Karsh et al., 2016, 2021; Tanaka et al., 2021). Accordingly, it was hypothesized to support developing and refining perceptual-motor representations (or ‘event files’) and facilitates responses to an associated stimulus (Hommel, 2022; Tanaka et al., 2021). The findings from the current study extend previous work on such motor enhancement from temporally contiguous action–effect by demonstrating a broader scope of its implications for response inhibition.

The work of Tanaka et al. (2021) described above may suggest how temporally contiguous action–effects can improve response inhibition through an automatic bottom-up process independent of additional cognitive control resources. Specifically, the work of Tanaka et al. (2021) suggests that the facilitating impact of an Immediate effect on motor response selection depends on a specific combination between the stimulus and the response, potentially enhancing selective stimulus–response association. Such enhanced stimulus–response association can be developed in the present study when a frequent Go stimulus is selectively associated with a particular response. According to such enhanced selective association hypothesis, the immediate perceptual action–effect (compared to the Lagged action–effect) improves the selective association between the Go response and the Go stimulus, reducing Go responses to other stimuli (e.g., the No-Go stimuli).

The SST (Experiment 2) findings also revealed an additional impact of temporally contiguous action–effect on motor performance that may challenge action control (e.g., as indicated by a higher error rate in Go trials and a lower rate of successful stopping after outliers exclusion, see Supplemental Materials). These findings suggest that an Immediate action–effect can also enhance general readiness to respond or modulate the response threshold (see also, Karsh et al., 2021), which may challenge action control. Note that these findings were only observed in Experiment 2 in the SST. Thus, further research is required to investigate whether such a higher error rate in Go trials and a potentially lower rate of inhibited responses following an Immediate action–effect is more likely when response inhibition must be accomplished by controlled (as in SST) rather than automatic (as in GNGT) means.

#### Study limitations

In Experiment 1, the rate of successful No-Go trials was relatively high, and the effect size of the contribution of temporally contiguous action–effect to No-Go performance was modest. These two aspects may result from the rather long trial duration (e.g., 1300 ms). Such a trial length was necessary to ensure participants experienced the lagged action–effect before the trial ended while keeping the trial duration equal in the two temporal contiguity conditions. However, previous work indicates that such trial duration is sufficiently short to generate a prepotent response (Wessel, 2018). Another explanation for the relatively modest effect size of response inhibition differences in Experiment 1 is that an action’s perceptual effect does not directly impact cognitive control but contributes to response inhibition by narrowing the activation of the Go response to a specific stimulus.

Another limitation of the current study stems from using different tasks to investigate different types of response inhibition. Specifically, in addition to the primary dependent variables measured in each task (No-Go performance versus SSRT), the tasks also differ in the probability of No-Go\Stop (20% vs. 25%) trials and the color\shape that differentiated Go from the No-Go\Stop trials. Although tasks’ parameters were accorded to previous recommendations for measuring response inhibition (Verbruggen et al., 2019; Wessel, 2018), a direct comparison of response inhibition between the two tasks is not optimal because of their possible different demands. Note that such a comparison was out of the scope of the current study, which focused on the impact of action–effect delay on response inhibition in each task separately. Future studies may benefit from using similar task designs to compare response inhibition between the tasks while minimizing possible interaction–effects stemming from different task parameters and action–effect delay.

Finally, the decision to base the initial data collection in Experiment 2 on a medium effect size (*d* = 0.6) could have been better, given the smaller effect size observed in Experiment 1 for the proportion of No-Go responses. Our commitment to the pre-registration drove this decision, which dictated a conclusive Bayes Factor as a stopping rule for Experiment 2. Nevertheless, it is essential to recognize that strict adherence to pre-registration may not always align with the optimal methodological approach for experimentation. These facets of the current research should be considered thoroughly in future studies.

#### Perspectives

The present study underscores the need for future research to explore the possibility that deficits in response inhibition performance may stem from issues in bottom-up sensorimotor processes or underdeveloped sensorimotor representations. This perspective suggests that the underlying causes of impaired response inhibition might differ between typical developmental trajectories and various clinical conditions, such as attention-deficit/hyperactivity disorder (ADHD), developmental coordination disorder (DCD), schizophrenia, compulsive disorders, and autism spectrum disorders. A deeper understanding of the specific sensorimotor and/or cognitive control dysfunctions contributing to response inhibition challenges in these conditions may help advance their diagnosis and treatment.

#### Conclusion

The present study demonstrates for the first time the contribution of action’s immediate perceptual effect to superior motor performance, as indicated by shorter response times and enhanced response inhibition (Experiment 1). The facilitating impact on Go-RT without modulating SSRT (Experiment 2) implies that such contribution operates at the sensorimotor level rather than improving cognitive control ability. The findings can be understood by the enhancing impact of an Immediate action–effect on the stimulus–response association, contributing to a selective activation of responses. The results also suggest an increase in general readiness to respond following an Immediate action–effect, which may hamper action control, at least when the task involves more controlled and less automatic processes (Experiment 2).

### Supplementary Information

Below is the link to the electronic supplementary material.

Supplementary file1 (DOCX 21 KB)

## Data Availability

Data and scripts are openly available from OSF repository at: https://osf.io/cr2ds/?view_only=184dc7b65cfb4b4bbb0cdab4066b8782
