# Supplementary material for: A differential impact of action–effect temporal contiguity on different measures of response inhibition in the Go\No-Go and Stop-signal paradigms
Source: Psychol Res. 2024 Mar 7;88(4):1157–68. doi: 10.1007/s00426-024-01931-2 (PMC11143021; doi:10.1007/s00426-024-01931-2)
Supplement: Supplementary file 1 — Supplementary file1 (DOCX 21 KB) [file 426_2024_1931_MOESM1_ESM.docx]

**Supplemental Materials**

A differential impact of action-effect temporal contiguity on different measures of response inhibition in the Go\No-Go and Stop-Signal paradigms.

N. Karsh, E. Soker-Mijalevich and O. Horovitz

**Table S1**. Experiment 1: Pearson correlations between self-report and behavioral measures in the Immediate and the Lag effect conditions.

|  | Go-RT  Immediate  Effect condition | Proportion of No-Go Responses  Immediate effect condition | Go-RT  Lag  Effect condition | Proportion of No-Go Responses  Lag effect condition | An urge to have an effect | Enjoyment | Invested effort | Trying to succeed in the task | Trying to be fast | Trying to be accurate | Mood |
| --- | --- | --- | --- | --- | --- | --- | --- | --- | --- | --- | --- |
| Go-RT | 1 |  | 1 |  |  |  |  |  |  |  |  |
| Proportion of No-Go Responses | .31** | 1 | .3* | 1 |  |  |  |  |  |  |  |
| An urge to have an effect | -.04 | .02 | -.11 | -.01 | 1 |  |  |  |  |  |  |
| Enjoyment | -.24* | -.18 | -.2 | -.04 | .02 | 1 |  |  |  |  |  |
| Invested effort | -.04 | -.08 | -.14 | -.17 | .13 | .14 | 1 |  |  |  |  |
| Trying to succeed in the task | -.03 | -.01 | -.20 | -.12 | .32** | .23* | .54** | 1 |  |  |  |
| Trying to be fast | -.38** | -.26* | -.32** | -.19 | .14 | .21 | .33** | .61** | 1 |  |  |
| Trying to be accurate | -.02 | -.06 | -.19 | -.08 | .41** | .11 | .29* | .53** | .39** | 1 |  |
| Mood | -.08 | .02 | -.14 | .04 | .2 | .46** | .14 | .13 | .04 | .15 | 1 |

The correlations are calculated for each condition separately.

*p*<.05, ***p*<.01

**Table S2**. Experiment 2: Pearson correlations between self-report and behavioral measures in the Immediate and the Lag effect conditions.

|  | Go-RT  Immediate  Effect condition | Proportion of stopping  Immediate effect condition | SSRT  Immediate effect condition | Go-RT  Lag  Effect condition | Proportion of stopping  Lag effect condition | SSRT  Lag effect condition | An urge to have an effect | Enjoyment | Invested effort | Trying to succeed in the task |
| --- | --- | --- | --- | --- | --- | --- | --- | --- | --- | --- |
| Go-RT | 1 |  | -.63** | 1 |  | -.57** |  |  |  |  |
| Proportion of stopping | .82** | 1 | -.83** | .5** | 1 | -.23 |  |  |  |  |
| SSRT |  |  | 1 |  |  | 1 |  |  |  |  |
| An urge to have an effect | -.08 | -.12 | .18 | -.3 | -.22 | .28 | 1 |  |  |  |
| Enjoyment | -.16 | -.13 | .07 | -.26 | -.05 | .11 | .82** | 1 |  |  |
| Invested effort | -.08 | -.13 | .19 | -.27 | -.17 | .31 | .93** | .76** | 1 |  |
| Trying to succeed in the task | -.09 | -.15 | .2 | -.28 | -.15 | .28 | .88** | .75** | .9** | 1 |

The correlations are calculated for each condition separately.

**p*<.05, ***p*<.01

*Experiment 2: Additional analyses excluding data from two outliers in SSRT*

Because the data included two outliers whose SSRT was lower than two standard deviations from both conditions’ mean, we conducted additional analyses without these outliers (this filtering criterion was not pre-registered).

The additional analyses revealed a lower rate of stopping performance in the Immediate (*M*=.48, *SD*=.01) than the Lag (*M*=.49, *SD*=.01) effect condition [*t*_29_=3.11, *p=*.004, CI_95_ (.002, .01), *dz*=0.56, BF_10_=9.71]. As in the primary analysis, the rate of correct Go trials was lower in the Immediate (*M*=.92, *SD*=.05) than in the Lag (*M*=.93, *SD*=.03) effect condition [*t*_29_=2.14, *p*=.04, CI_95_ (.00, .02), *dz*=0.39, BF_10_=1.4]. Similar to the primary analyses, Go-RT was shorter in the Immediate (*M*=443, *SD*=38) compared to the Lag (*M*=464, *SD*=38) effect condition [*t*_29_=3.72, *p*<.001, CI_95_ (9, 32), *dz*=0.68, BF_10_=38] and SSD was shorter in the Immediate (*M*=187, *SD*=41) compared to the Lag (*M*=214, *SD*=46) effect condition [*t*_29_=3.84, *p*<.001, CI_95_ (12, 40), *dz*=0.7, BF_10_=51]. Importantly, there was still no significant difference in SSRT between the Immediate (*M*=255, *SD*=17) and the Lag (*M*=250, *SD*=21) condition [*t*_29_=1.35, *p*=.18, CI_95_ (-2, 12), *dz*=0.24, BF_10_=0.44].
